# Control Measurements of *Escherichia coli* Biofilm: A Review

**DOI:** 10.3390/foods11162469

**Published:** 2022-08-16

**Authors:** Feng Zhou, Dehua Wang, Jiamiao Hu, Yi Zhang, Bee K. Tan, Shaoling Lin

**Affiliations:** 1 Engineering Research Centre of Fujian-Taiwan Special Marine Food Processing and Nutrition, Ministry of Education, Fuzhou 350002, China; 2 Department of Cardiovascular Sciences, Leicester Diabetes Centre, College of Life Sciences, University of Leicester, Leicester LE1 7RH, UK; 3 Diabetes Research Centre, Leicester General Hospital, Leicester LE5 4PW, UK

**Keywords:** *Escherichia coli biofilm*, food industry, sterilization, control measurements

## Abstract

*Escherichia coli* (*E. coli*) is a common pathogen that causes diarrhea in humans and animals. In particular, *E. coli* can easily form biofilm on the surface of living or non-living carriers, which can lead to the cross-contamination of food. This review mainly summarizes the formation process of *E. coli* biofilm, the prevalence of biofilm in the food industry, and inhibition methods of *E. coli* biofilm, including chemical and physical methods, and inhibition by bioactive extracts from plants and animals. This review aims to provide a basis for the prevention and control of *E. coli* biofilm in the food industry.

## 1. Introduction

*Escherichia coli* (*E. coli*), a member of *Enterobacteriaceae*, was discovered by Escherich in 1885. It is 0.5 × 1-3 μm in size with peritrichous flagellae [1]. *E. coli* can also ferment a variety of sugars to produce acid and gas. Certain special serotypes of *E. coli* were recognized as pathogenic and capable of causing human- and animal-infections by the mid of 20th century. In 1982, *E. coli* was first defined as an intestinal pathogen and an important source of food-borne diseases. These bacteria can cause serious diseases, such as hemorrhagic proctitis, hemolytic uremic syndrome, and acute renal failure. Additionally, *E. coli* can easily form biofilms, which also affects human health [2,3].

At present, despite plenty of research for strengthening the prevention and control of *E. coli*’s pollution, the incidents of food poisoning by the contamination of *E. coli* are still common, and have caused serious damage to human health, as well as huge losses to the food industry and economy [4]. In 2018, there was an *E. coli* outbreak in 13 states in North America, which caused multiple infections [5]. In 2011, Germany experienced the spread of enterohemorrhagic *E. coli* (EHEC) disease. Consumers ate contaminated bean sprouts, which caused diarrhea, hemolytic uremic syndrome and even death [6,7]. In 1996, a large outbreak of *E. coli O157* food poisoning occurred in dozens of middle schools and kindergartens in Okayama, Hiroshima, and other areas of Japan, and the number of infections reached nearly 10,000 [8].

Notably, the biofilm-formation capability of *E. coli* can greatly increase its resistance to environmental stress and often results in sterilization failures. Biofilm is composed of microbial colonies and self-produced extracellular polymeric substances (EPS), where the microorganisms of the same or different species are spontaneously wrapped in their self-secreted EPS matrix, which provides resistance to the external environment, and attachment to the surface of living or non-living carriers [9,10,11]. Therefore, the purpose of this article is to briefly review the prevalence, formation process, and control measures (chemical, physical, biological components) of *E. coli* biofilm, which could provide novel insights into the control of *E. coli* biofilm in the food industry.

## 2. Stages of the Formation of *E. coli* Biofilm

The formation of microbial biofilm is a universal phenomenon. As long as the conditions permit, microorganisms often form biofilm in the environment [12]. The formation of biofilm depends upon the changes in nutrients, temperature, osmotic pressure, pH, and other factors in the environment. The formation of biofilm provides protection for microorganisms. The microorganisms in biofilm exhibit stronger environmental resistance than the migratory microorganisms and can survive in harsh environmental conditions.

**Figure 1:**
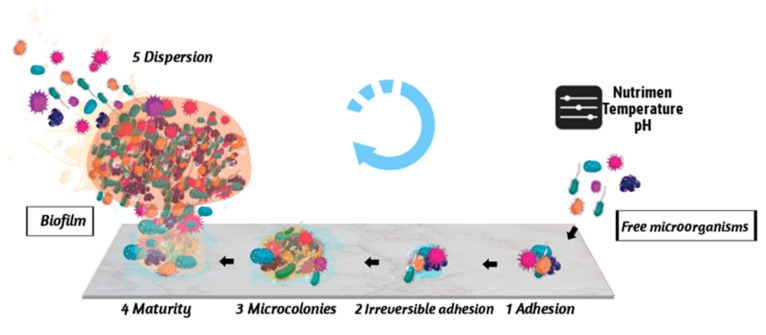
Schematic diagram of microbial biofilm formation. When the environmental conditions (nutrients, temperature, pH, etc.) are adverse, the free microorganism actively and reversibly adheres to the carrier surface, and then secretes EPS (blue) to further enhance the adhesion between the microorganism and the carrier by forming irreversible adhesion. Then, the microorganism grows and propagates to form colonies, and biofilm (yellow) is formed on the surface. After the increase in microorganisms, the biofilm is mature, becoming mushroom-like and with a three-dimensional structure. With the increase in microorganisms in the biofilm, they disperse from the biofilm to form new free microorganisms. As the cycle continues, microorganisms can survive in harsh environments.

Since the observation of microbes and biofilms by Antony van Leeuwenhoek in dental plaques using a microscope in 1676, studies about the mechanisms of biofilm formation have evolved rapidly [13]. To date, the formation of *E. coli* biofilm is generally divided into five stages, including reversible adhesion, irreversible adhesion, colony formation, biofilm maturation, and dispersion, as shown in Figure 1.

### 2.1. Reversible Adhesion between Microorganisms and Carriers

The free microorganisms receive environmental signals (such as nutrients), and use their extracellular organelles (such as flagella, cilia, and curly fimbriae) and outer membrane proteins in order to adhere to the carrier surface.

### 2.2. Irreversible Adhesion between Microorganisms and Carriers

Microorganisms secrete EPS, which enhances the adhesion of microorganisms with the carrier surface. EPS is composed of nucleic acids, proteins, lipids, lipopolysaccharides, and other substances, and forms a protective barrier for microorganisms by restricting and preventing the antibacterial agents from reaching the biological membrane of target organisms [14,15]. Notably, the latest findings also reveal that the bacterial organelles play important roles in the microbial irreversible attachment during the biofilm formation. For instance, the bacterial cell wall often deforms at this stage, which could enhance the adhesion between microorganisms and carriers [16].

### 2.3. Colony Formation Stage

The microorganisms, adhering to the carrier surface, grow and multiply to form colonies. Then, the continuous proliferation of microorganisms makes their biological growth space crowded with a lack of nutrients and accumulation of toxic substances. At this stage, the quorum-sensing (QS) signals, a cell-to-cell communication mechanism in bacteria, could induce the *E. coli* into the biofilm lifestyle by regulating the synthesis of biofilm matrix compounds [17]. During this process, EPS is significantly increased, forming a gel phase on the surface of the carrier to protect the microorganisms [18].

### 2.4. Biofilm Maturation Stage

*E. coli* microcolonies continue to accumulate, which further increases the thickness of the film and results in the formation of mushroom-like or columnar subunits. Then, the biofilm grows in a three-dimensional manner and eventually forms a viable three-dimensional structure [19].

### 2.5. Biofilm Dispersion Stage

The dispersion of biofilm can be divided into active dispersion and passive dispersion. The active dispersion refers to a decrease in the level of cyclic diguanylate (c-di-GMP) in cells, which results in the production of enzymes that degrade the matrix of biofilm and promote its dispersion. The passive dispersion depends on the external factors, such as physical triggers (wear, fluid shear, etc.) and enzymatic degradation (extracellular hydrolase) [20]. The dispersion of microorganisms from biofilm is beneficial for their reproduction and biofilm recombination, thereby forming a cycle of wandering microorganisms and biofilm [12].

In conclusion, the formation of *E. coli* biofilm is a strong survival support for *E. coli* in unfavorable living environmental conditions, protects the growth and reproduction of *E. coli* to the greatest extent, and provides favorable conditions for *E. coli* to wreak havoc on and pollute food, eventually causing infection and affecting human health.

## 3. Prevalence of *E. coli* Biofilm in the Food Industry

Hygienically, *E. coli* is an indicator of fecal pollution, and a serious threat to the life and health of consumers. Once the content of *E. coli* in a food item exceeds the standard, it is withdrawn from the shelves, resulting in huge economic losses.

Moreover, the biofilm-making ability of *E. coli* may pose a greater threat to the processing and production of food. Studies show that once the microbial biofilm is formed, the resistance of bacteria to disinfectants is increased by 500 times, suggesting that, in the presence of biofilms, the germicidal time and concentration of disinfectants must be increased to 10–100 times to kill bacteria [21,22]. Another study also proposes that the EPS of biofilm can inhibit antibiotics and bactericides from entering cells, and reduce the permeability, resulting in a significant increase in the concentration of antibiotics and bactericides in the presence of biofilm [23].

At present, a couple of studies have shown that the *E. coli* biofilm exists in nearly all aspects of the processing and production of all kinds of food (brewing industry, dairy products, fresh food, meat products, etc.), and may cause pipeline biological pollution and equipment damage (water system, cooling tower, and other technical failures), thereby contaminating food items [18,24,25,26,27].

Food ingredients can also be easily contaminated by *E. coli*, as it grows in a natural environment. This may introduce *E. coli* into the food industry. Studies have reported the isolation of *E. coli* from peppers, tomatoes, cantaloupes, and other crops in Nuevo Leon and Coavira, Mexico. A total of 341 strains of *E. coli* have been isolated, of which 76% of strains have the ability to form biofilms. Among them, 34% of *E. coli* strains form strong biofilms [5], which pose a potential risk to endanger consumers’ health. At the same time, due to the pathogenicity of *E. coli* to cause human and animal diseases, a great importance has been given to the detection of *E. coli* in poultry products. At present, Brazil is the largest exporter of chicken. Among the 88 strains of *E. coli* isolated from the chicken meat of four retail chicken companies in Brazil, 31 strains could form a strong biofilm, while only four strains could not form biofilm [28]. In addition, *E. coli* can easily form biofilms in processing equipment, such as stainless-steel metal products [29,30], polyethylene [31], walls and drains [32], due to improper cleaning. Nancy et al. isolated *E. coli* from fresh-cut processing equipment. The co-culturing of *E. coli* with the isolated *Burkholderia caryophylli* and *Ralstonia insidiosa* increases biofilm biomass by 180% and 63%, respectively, posing a great challenge to eliminating the foodborne pathogens in fresh-cut processing equipment [33].

## 4. Control Measures for the Prevention of *E. coli* Biofilm Formation

*E. coli* can form biofilm on the surface of food items, pipes, and processing equipment, which greatly increases its resistance to environmental conditions and reduces the effectiveness of disinfectants [34]. The biofilm increases the possibility of cross-infection of *E. coli* in food items and harms the health of consumers. Therefore, it is urgent to take effective measures to reduce or control the formation of *E. coli * biofilm during processing, as shown in Table 1, to reduce the risk of microbial contamination.

### 4.1. Chemical Methods

At present, the disinfectants, commonly used in food processing and slaughterhouses, include chlorine disinfectants, quaternary ammonium chloride (QAC), and lactic acid, which inhibit the formation of *E. coli* biofilm to a certain extent, but cannot completely inactivate it [56]. When studying the inhibitory effects of chlorine dioxide (CD), neutral oxygen potential water (NEOW), and sodium hypochlorite (SH) on *E. coli* biofilm, the inhibitory effects of NEOW and CD on *E. coli* biofilm biomass were significantly higher than that of SH and exhibited a certain degradation effect on *E coli* biofilm. Among them, NEOW was the most stable at a low temperature (5 °C) with the lowest chlorine loss rate and a wider bacteriostatic temperature range [35]. The comparative study of the inhibitory effect of 2-hydroxypropyl-3-piperazinyl-quinoline carboxylic acid methacrylate (HPQM) and silver-substituted zeolite (ZEOMIC) on linear low-density polyethylene (LLDPE) *E. coli* biofilm suggested that the HPQM exhibited comparatively better inhibitory properties and bacteriostatic effects on LLDPE with low roughness than ZEOMIC [36]. At the same time, a study has shown that different materials can affect the effectiveness of disinfectants. For example, ClO_2_ and SH were found to show the highest anti-biofilm activity against *E. coli* on stainless steel, followed by glass, plastic, and wood [37]. This information is useful in food processing for the selection of correct bactericides according to the actual application and different contact surfaces in food processing. However, a study demonstrated that the SH and lauroyl arginate ethyl (LAE) could inhibit the biofilm of *E. coli* on the surface of Hami melon, but a 2000-μg/mL concentration of SH and arginine LAE had no obvious effects on the inhibition of *E. coli* biofilm, and the biofilm showed high resistance to disinfectants [38]. Therefore, for the conventional chemical disinfectants, it is difficult to penetrate and spread into the biofilm in order to kill microorganisms and can no longer meet the standards of controlling microorganisms in food processing. In addition, using chemical methods for microbial control, such as chemical preservatives, not only easily produces adverse effects on food sensory properties and nutrition, but also easily causes allergies and other problems. Therefore, there is an urgent need to find new antibacterial methods or antimicrobial agents to reduce the occurrence of microbial cross-contamination. With the deepening of the research on microbial sterilization, anti-microbial photodynamic technology, to a certain extent, makes up for the shortcomings of general chemical bactericides, and shows great advantages in the pharmaceutical and food industries. A study has shown that riboflavin-mediated photodynamic sterilization technology has a significant inhibitory effect on *E. coli* biofilm and the mixed biofilm of *E. coli* and *Staphylococcus aureus*. Its inhibitory effect is mainly through the rise of reactive oxygen species, leading to bacterial oxidative stress and damaging the respiratory system [39].

### 4.2. Physical Methods

For the inhibition of *E. coli* biofilm in food processing and production, physical methods (washing, high-temperature sterilization, etc.) are often used in addition to chemical methods to reduce the occurrence of food’s cross-contamination. High-temperature sterilization is the most common physical method to control microorganisms. Saturated steam (SS) and superheated steam (SHS) can inactivate polyvinyl chloride (PVC) in food processing facilities and inhibit the formation of *E. coli* biofilm on the surface of stainless steel. SHS showed higher lethality and better inactivation of the cells in *E. coli* biofilm [40]. To prevent the formation of bacterial biofilm, it is a good method to suppress the biofilm adhesion on a solid surface by vibration. A study has shown that nano-vibration on the surface of materials can effectively inhibit the formation of *E. coli* biofilm. When the amplitude of vibration on the surface is greater than 21nm, the greater the amplitude, the less the number of viable bacteria. It can provide a reference for some precise and difficult methods to clean mechanical surfaces in food or medicine [57]. In addition, new physical sterilization technologies, including low-temperature plasma sterilization, not only show good anti-biofilm activities, but also retain the original sensory properties of food items to the greatest extent [58]. The low-temperature plasma sterilization also showed a good inhibitory effect on the formation of *E. coli* biofilm and a reduction in *E. coli*’s abundance under the biofilm to 5log_10_. At the same time, the low-temperature plasma sterilization, in combination with water spray, can not only inhibit bacteria, but also improve the efficiency of antifouling [41]. Similarly, with the extension of the study, Kadri et al. also found that cold atmospheric plasma treatment with a flow rate of 5 L/min could significantly inhibit *E. coli* biofilm and *Listeria* biofilm, while this inhibitory effect slightly decreased as biofilm growth age increased. Afterwards, a mixed culture of the two bacteria showed that the production of EPS in *E. coli* biofilm may contribute to the increases in the resistance of cold atmospheric plasma, which provides a new target to inactivate bacteria on the complex solid surface [59]. In addition, Adator et al. found that when the temperature was lower than 10 °C, *the Shiga toxigenic E. coli* (STEC) in the biofilm on stainless steel would not transfer to lettuce. However, when the temperature is 25 °C, STEC will transfer from the biofilm to stainless steel. It is suggested that keeping a low temperature in a food processing environment is helpful to control the transfer of *E. coli* in biofilm, resulting in microbial cross-contamination [60].

In the actual food processing and production, food industries often use the combination of chemical and physical sterilization methods to control microorganisms, and the synergistic effect between them maximizes the removal or inhibition of *E. coli* biofilm. The combination of lactic acid (LA) with water vapors can effectively reduce the abundance of *E. coli* on the surface of PVC and stainless steel, and exhibits lethal effects on the microorganisms in biofilm [42]. The combination of cold nitrogen plasma (CNP) with clove oil also showed significant synergistic inhibitory effects on *E. coli* biofilm [43].

### 4.3. Biological Components

In recent years, due to the decrease in the sensitivity of microorganisms in biofilm to conventional disinfectants, the germicidal efficacy of conventional disinfectants cannot reach an ideal state, which increases the risk of food microbial safety. Therefore, there is an urgent need to seek new active germicidal substances and improve their efficiency. Biological extracts are natural compounds, which avoid the problem of chemical disinfectant residues and have attracted wide attention because of their higher level of safety. Therefore, the inhibitory effects of biological extracts on *E. coli* biofilm have been widely reported.

With the attitude of safety, environmental protection, economy, and turning waste into treasure, animal extracts such as the scavenging effects of scallop shells on *E. coli* biofilm have attracted the attention of researchers. The main component of scallop shell is calcium carbonate (CaCO_3_), which is easily decomposed into calcium oxide (CaO) by heating, which then exhibits broad-spectrum antibacterial properties [61,62]. Studying the scallop shell powder (SSP) for its anti-biofilm activity, it was found that only 0.25% and 0.5% of scallop shell powder could inhibit *E. coli* biofilm on the surface of stainless steel in whey powder solution and meat processing plant washing water by 4 and 6 log CFU/cm^2^ and 3 and 5 log CFU/cm^2^, respectively. This inhibitory effect was positively correlated with the concentration of scallop shell powder, suggesting that it can be a good candidate for application in the food industry [44].

In addition, the concept of using bacteriophages, the natural predators of bacteria, as a novel strategy to prevent and eliminate biofilms has gradually attracted the attention of researchers. The treatment with phage AZO145A significantly inhibited the biofilm formation of STEC on the surface of stainless-steel plate, and significantly inhibited their migration at 24 °C in the biofilm formed in beef, which was reduced to 3.1 log_10_ CFU, thereby reducing the risk of food cross infection [45]. At the same time, with the in-depth study of bacteriophages, increasingly more bacteriophages were found to have a good inhibitory effect on *E. coli* biofilm, such as bacteriophage FP43, Daica, and135 [27,46]. In the bacteriostatic effect of phage, phage cocktails have a better inhibition effect on biofilm than that of single phage. The main inhibition process is to decompose biofilm into weak biofilm or non-biofilm to reduce bacteria [47].

The inhibitory effects of plant extracts on the biofilm of *E. coli* have been a hot topic. The plant extracts can be directly used in food processing equipment or food items to inhibit the *E. coli* biofilm and prolong the shelf-life of food items. Phenols are widely studied for their inhibitory effects on *E. coli* biofilm. Four components were obtained after extracting the active components from burdock leaves with ethanol and diluted with water to form different concentrations of ethanol (20%, 34%, and 70%). It was found that the active components obtained by different ethanol elution could significantly inhibit the *E. coli* biofilm, where the inhibitory effects were correlated with the phenol contents in the burdock leaves. The 70% ethanol elution could seriously damage the structure of *E. coli* biofilm, which no longer showed the state of a multi-layer growth [48]. The comparative study of nine catechol compounds, including catechol (CAT); veratrylalcohol (VER); guaiacol (GUA); 2-ethoxyphenol (ETH); 4-methylcatechol (MEC); 4-tert-butyl catechol (TEB); pyrogallol (PYR); 3-methoxycatechol (MET); and o-phenylene-phosphochloridite (OPP), suggested that the ETH, MEC, TEB, and PYR had the best inhibitory effects on *E. coli* biofilm [49]. The low-concentration carvanol revealed significant inhibitory effects on *E. coli* biofilm, which were proportional to the time, and could completely remove the biofilm after 15 min of exposure [50]. The characteristics of carvanol, such as low concentration and long time, can meet the requirements of disinfection in the food industry. Similarly, 50 μg/mL of coumarin could also inhibit more than 80% of *E. coli* biofilm without affecting the growth of bacteria [51]. In addition, the clove oil, lectins, and other substances also showed inhibitory effects on *E. coli* biofilm. The combination of clove oil and liposome, making solid liposomes (SLPs), can avoid the disadvantage of volatilization under light and at a high temperature, and improve stability and the utilization rate. The SLPs treatment dispersed the *E. coli* biofilm from the whole to a loose state, which seriously damaged its structure, decreased the biomass under membrane, and saw some cells collapse. In addition, the application of SLPs to the surface of cucumber and lettuce also showed a good inhibitory effect on biofilm and prolonged the shelf life of vegetables [52]. The antibacterial lectin, extracted from pomegranate peel, was found to have inhibitory effects on all the drug-resistant *E. coli* biofilms, with an inhibition rate of 50% at 6.25 g/mL or more [53].

The inhibitory effects of plant extracts on *E. coli* biofilm mainly depend on the inhibition of cell metabolic activities; gene expression; reduction in extracellular polysaccharides, bacterial motility, and cell hydrophobicity; and directly preventing the initial adhesion stage of biofilm formation. The treatment of *E. coli* with SLPs suggested a negative correlation of the SLPs concentration of clove oil with the bacterial metabolic activities, extracellular polysaccharides, and protein contents, thereby weakening the protective barrier and adhesion of bacteria [52]. The extract of *Coptis chinensis* could also inhibit the metabolic activities of *E. coli* biofilm [54]. Coumarin, umbelliferone, and aescin significantly inhibited the expression of CSG operon, involved in curl formation, and movement genes; reduced the formation of fimbriae; prevented the adhesion of *E. coli* to the surface; and inhibited the formation of biofilm [51]. Studying the inhibition of *E. coli* biofilm with triterpenoids (oleanolic acid and ursolic acid) suggested that oleanolic acid analogues could change the expression of genes involved in the regulation of type IV pili [63]. CAT, TEB, and PYR could significantly reduce the mobility of *E. coli* by inhibiting the formation of biofilm, and ETH, MEC, TEB, and PYR could significantly reduce the surface hydrophobicity of *E. coli* cells and hinder the adhesion stage during the formation of *E. coli* biofilm [49]. In the inhibition of vitamin C on *E. coli*, vitamin C can down-regulate the signal transduction genes and regulatory genes of biofilm by more than 27 times [4]. In the study of the inhibitory effect of cinnamon, marjoram, and thyme on the biofilm of *E. coli* and *Listeria* on the surface of polypropylene, it was also found that the inhibitory effect of cinnamon, marjoram, and thyme could be achieved by penetrating the biofilm and cell membrane, further changing the fluidity and permeability of the membrane, condensing the protons of the cytoplasm to form the weak mitochondria [55].

## 5. Conclusions

The formation of microbial biofilm is a kind of self-protection behavior of microorganisms in unfavorable environmental conditions, which helps them in avoiding environmental stresses. Therefore, the *E. coli* biofilm has become a hidden danger of microbial contamination in the food industry. The formation of *E. coli* biofilm is a complex process, which is affected by a variety of regulatory networks. At present, there is an urgent need for studies on the regulatory mechanism of biofilm maturation, as well as the prevention methods of biofilm formation. Meanwhile, studying the mechanisms of the inhibition of *E. coli* biofilm is of great significance for the development of efficient and safe germicide to reduce *E. coli* biofilm in the food industry.

## Figures and Tables

**Table 1:** Inhibition effect of different sterilization methods on *E. coli* biofilm.

Bacteriostatic Mode	Bacteria	Carrier Material	Antibacterial Substance	Concentration	Process Time	Reduction (logCFU·cm^−2^) or Bacteriostatic Rate	Reference
Chemical methods	*E. coli CECT 434*	Stainless steel AISI 316	Neutral oxygen potential water	50 ppm	20 min	3.26	[35]
			Chlorine dioxide	50 ppm	20 min	3.20	
			Sodium dichloroisocyanurate	50 ppm	20 min	3.20	
			Sodium hypochlorite	50 ppm	20 min	2.46	
	*E. coli ATCC 25922*	Linear low-density poly-ethylene	2-hydroxypropyl-3-piperazinyl-quinoline carboxylic acid methacrylate	1500–2500 ppm	3-5 day	99%	[36]
	*E. coli O157:H7*	Stainless steel	Sodium hypochlorite (NaOCl)	200 μg/mL	15 min	7.7	[37]
			Aqueous chlorine dioxide (ClO_2_)	200 μg/mL	15 min	ND	
		Glass	NaOCl	200 μg/mL	15 min	8.2	
			ClO_2_	200 μg/mL	15 min	ND	
		Plastic	NaOCl	200 μg/mL	15 min	3.3	
			ClO_2_	200 μg/mL	15 min	ND	
		Wood	NaOCl	200 μg/mL	15 min	1.3	
			ClO_2_	200 μg/mL	15 min	1.5	
	*E. coli O157:H7 B6-914*	Glass cover Slides	Lauroyl arginate ethyl	200 μg/mL	24 h	0.46	[38]
			Sodium hypochlorite	200 μg/mL	24 h	0.59	
	Multi drug resistant *Escherichia coli*	glass slides	Photodynamic antimicrobial chemotherapy	50 μM	10min	34%	[39]
Physical methods	*E. coli O157:H7*	Polyvinyl chloride	Saturated steam	/	5 s	1.21	[40]
			Superheated steam	/	5 s	1.26	
		Stainless steel	Saturated steam	/	5 s	1.52	
			Superheated steam	/	5 s	1.84	
	*E. coli strain BW25113 F+*	Glass cover slides	Positive corona	/	15 min	5.28	[41]
			Negative corona			5.4	
	*E. coli O157:H7* *(ATCC 35150,* *ATCC 43889,* *ATCC 43890)*	Polyvinyl chloride	Lactic acid and water vapors	0.5%–2%	5 s	0.76–3.78	[42]
		Stainless steel	Lactic acid and water vapors	0.5%–2%	5 s	1.64–3.92	
	*E. coli EHEC O157:H7 CICC 21530*	Stainless steel	Clove oil	1 mg/mL	30 min	3.32	[43]
			Cold nitrogen plasma	/	3 min	2.23	
Biological components components	*E. coli O157:H7 NCTC 12900*	Stainless steel	Scallop shell powder	0.25%	1 min	4-6	[44]
				0.5%		3-5	
	*STEC O145:H25*	Stainless steel	Bacteriophage AZO145A	2 × 10^10^ pfu/mL	3 h	3.1	[45]
	*Escherichia coli O157:H7 and O91:H-*	96-well plates	Bacteriophage FP43	10^10^ pfu/mL	6 h	2.85	[46]
	*E. coli O177*	96-well polystyrene plates	Phage cocktail stock	1 × 10^8^ pfu/mL	24 h	ND	[47]
	*E. coli ATCC25922*	Silicone disks	Components of burdock leaves	0.017 mg/mL	24 h	50%	[48]
	*E. coli CECT434*	96-well microtiter plates	2-ethoxyphenol	7 mM	24 h	58.0 ± 15.0%	[49]
			4-methylcatechol	3.5 mM	24 h	61.0 ± 10.0%	
			4-tert-butyl catechol	1.6 mM	24 h	77.0 ± 0.0%	
			pyrogallol	5 mM	24 h	73.0 ± 4.0%	
	*E. coli* *O157:H7 ATCC 35150*	Stainless steel	Carvacrol	1%	5 min	6.04	[50]
	*E. coli O157:H7 ATCC43895*	96-well polystyrene plates	Coumarins	50 µg/mL	24 h	above 80%	[51]
	*E. coli O157:H7*	48-well plate	Solid liposomes	0.5 mg/mL	24 h	65.74%	[52]
	*E. coli 2011-60-1493-3, 2011/25/62, C24716 C-26036*	96-well microtiter plate	Punica granatum sarcotesta lectin	≥6.25 g/mL	24 h	≥50%	[53]
	*Escherichia coli (ATCC 35218)*	96-well poly- styrene microtiter plate	Ethanol extract of Carum coptis chinensis	25 mg/mL	24 h	≥70%	[54]
	*E. coli EMC17*	96-well polystyrene plate	Vitamin C	30 mM	24 h	50%	[4]
	*Escherichia coli SZMC 0582*	Polypropylene spatula	Cinnamomum Zeylanicum	1.2 mg/mL	10 min	9 ± 5.45%	[55]
			Origanum majorana	4.5 mg/mL	10 min	100 ± 0.00%	
			Thymus vulgaris	3.8 mg/mL	10 min	100 ± 0.00%	
			HC-DPE (active ingredients: 15% peracetic-acid, 20% total peroxide)	0.1%	10 min	100 ± 0.00%	
			Sodium hypochlorite	0.84%	10 min	100 ± 0.00%	

ND: below the detection limit, not detected.

## Data Availability

Not applicable.
